# Household ownership and use of insecticide-treated bednets among school children in Ibadan, Oyo State, Nigeria

**DOI:** 10.5281/zenodo.10818068

**Published:** 2016-07-03

**Authors:** Justina U. Onwuka, Joshua O. Akinyemi, IkeOluwapo O. Ajayi

**Affiliations:** 1 Department of Epidemiology and Medical Statistics, College of Medicine, University of Ibadan, Oyo State, Nigeria

## Abstract

**Background:**

In or der to combat the bur den of malaria, different strategies, including Insecticide Treated Nets (ITNs), have been put in place. ITNs have been distributed with support from international donors and this necessitates an increase in monitoring and evaluation efforts in order to determine ITN impact as well as prioritise future programmes. The current standard for estimating impact indicators of ITNs are household surveys. These, however, are expensive and not conducted frequently. Collecting information from school children has been found to be a cheap and fast means for routine monitoring and evaluation of malaria control programmes in sub-Saharan Africa. This study was conducted to explore school children’s report of household ownership and use of ITNs in Oyo State, Nigeria.

**Materials and methods:**

A cross-sectional survey was conducted. A three-stage sampling technique was used to select 611 pupils from 15 out of 88 primary schools. Information on pupils’ socio-demographics, report of household ownership and use of ITNs were obtained using a semi-structured interviewer-administered questionnaire. Data was analysed using descriptive statistics and Chi-square tests at 5% level of significance.

**Results:**

Respondents’ mean age was 10.5±1.7 yrs; 52.7% were females, 84.6% were Yoruba and 65.3% lived with children below 5 yrs of age in their households. Most of the respondents (81.7%) reported household ownership of at least one ITN. The majority (76.4%) obtained nets through mass distribution campaigns. Most of the respondents (89%) reported use of ITNs by a household member the night preceding the survey. More than half of the respondents (51.6%) reported ITN use by children below 5 yrs of age. Class was significantly associated with reported household ownership of ITNs (χ2= 9.217, p <0.010).

**Conclusion:**

The majority of the pupils reported household ownership and use of ITNs. They should be consider ed a potential source of information in monitoring and evaluation activities related to ITN ownership and use.

## 1 Introduction

Malaria remains a major public health problem globally, with highest morbidity and mortality in sub-Saharan Africa, and is endemic in Nigeria, with year-round transmission [1]. Children below five years of age and pregnant women are most vulnerable to malaria [2]. In order to combat the disease, several strategies that include vector control (indoor residual spraying (IRS), insecticide-treated nets (ITNs)), prompt and effective treatment, and intermittent preventive treatment [2] have been put in place. ITNs have been found to be highly effective in protecting those sleeping under them and those nearby. ITNs have also been shown to reduce under-five mortality by about 20% in malaria-endemic areas in sub-Saharan Africa [3]. With support from international donors, ITNs have been distributed following the target set at the Abuja summit of achieving at least 60% coverage among vulnerable populations at risk of malaria [4], as well as to scale up to universal coverage for all the people at risk for malaria.

The scale-up in ITN delivery necessitates an equivalent increase in monitoring and evaluation (M&E) efforts in order to determine the impact of ITN distributions as well as prioritise future programmes [5]. Following the recent achievements in global malaria control, there is increased emphasis on monitoring these achievements so as to determine intervention needs [6] as well as assess their effectiveness and coverage [7]. But there are no health information systems that routinely monitor malaria control programmes including insecticide-treated nets coverage [8]. The current standard for M&E of ITNs are household surveys such as Demographic and Health Surveys (DHS) and Malaria Indicator Surveys (MIS). These are, however, expensive, not conducted frequently enough to provide annual estimates of ITN coverage for all countries [2], time consuming, technically complicated to undertake and usually provide information for national intervention coverage thereby requiring important human and financial resources [9]. Previous studies in some African countries have shown that collecting information from school children can be a complementary, inexpensive framework for planning and M&E of malaria control programmes [2,10,11] as well as ivermectin treatment coverage [12].

To provide a potential complementary medium for M&E of ITNs ownership and use in Nigeria, we obtained information on household ownership and use of ITNs from school children in Akinyele Local Government, Oyo State Nigeria.

## 2 Materials and methods

### 2.1 Study area

The study was conducted in Akinyele Local Government Area (LGA) of Oyo State, Nigeria. Akinyele LGA is made up of an estimated population of 211,359 and twelve geopolitical wards namely Ikereku (ward 1), Labode/Oboda/ Olanla/ (ward 2), Arulogun (ward 3), Onidundu/ Amosun (ward 4), Moniya (ward 5), Akinyele (ward 6), Iwokoto/ Amosun (ward 7), Ojoo/Ajibode/Orogun/Owe/Kankon (ward 8), Alabata (ward 10), Okegbemi/Mele (ward 11), Iroko (ward 12). Akinyele LGA is urban and rural; the population is dominated by the Yoruba ethnic group. The people of Akinyele are mainly traders and artisans. The area experiences a tropical climate with a mean annual temperature of 32^o^C. The relative humidity can be as high as 95% and rainfall totals 1250 mm per annum. The area is located in the forest belt of the country, particularly in the tropical rain forest. There were seventy eight (78) are private primary schools and one hundred and twenty three (123) public primary schools registered with the State Ministry of Education in Akinyele LGA. A total of 132,251 Long Lasting Insecticide-treated Nets (LLINs) were distributed in Akinyele LGA about six months prior to the study, through the universal net campaign organised by the National Malaria Elimination Programme of the Federal Ministry of Health (FMoH). Each household in the LGA received two nets (Oyo State Ministry of Health).

### 2.2 Study design

This study was a descriptive cross-sectional school-based survey. Pupils in primary 4 to 6 between the ages 7 to 13 years who were present in school on the day of the visit were recruited into the study. Pupils in primary 4 to 6 whose relations in any of the other classes were already participating in the study were excluded from the study. A sample size of 604 was calculated using the proportion of reported household ownership of at least one ITN by school children as 23% [7], with 5% level of precision, 95% confidence level, 10% contingency for non-response, assuming a design effect of two.

Akinyele LGA was conveniently selected since it is one of the LGA’s in Oyo State where ITNs have been distributed prior to the survey. A multi-stage sampling technique was used to select the wards, schools and respondents that participated in the survey. Three wards (wards 3,5 and 8) were selected from the existing twelve (12) wards using simple random sampling. Fifteen (15) schools were proportionately selected from the three wards. From each school, the pupils were stratified into primaries four, five and six (4, 5 & 6), then proportionate allocation of the sample size was used to determine the number of pupils that were selected from each stratum. Systematic random sampling was used to select the respondents that participated from each class.

### 2.3 Data collection and analysis

A semi-structured interviewer-administered questionnaire was employed in this study and used by trained interviewers. Data collection took place between October and November 2014. The questionnaire used was adapted from past surveys conducted in Nigeria [1,13] and was modified after it was pre-tested. The questionnaires were completed during school hours and were administered only to the pupils who gave verbal consent after explaining the purpose of the research, time that would be spent and the benefits of the research.

Data entry and statistical analysis were conducted using SPSS version 20 software. Descriptive statistics such as percentages, frequency counts, mean, and standard deviation were used to summarise the data and Chi square tests were used to investigate associations at 5% level of significance.

### 2.4 Ethical considerations

Ethical approval to carry out the study was obtained from the Oyo State Ethical Review Committee, Ministry of Health. Permission was also obtained from the Oyo State Universal Primary Education Board before proceeding to the schools that participated in the study. The Heads of the different schools that participated in the study stood as proxy for their parents and gave consent. Participation of the pupils was voluntary and those who decided to withdraw during the study were permitted to do so. The research did not cause any form of harm to the pupils and was conducted at a time that was convenient for them, not affecting their studies. Confidentiality of the information given by the pupils was ensured. Serial numbers were written on each questionnaire and no names were required from the participants.

## 3 Results

Table 1 shows the socio-demographic characteristics of the respondents. Respondents’ age ranged from 7-13 years with a mean age of 10.5 ± 1.7 years and the majority of the respondents (73.6%) were between the ages 10-13 years. Three hundred and twenty two (52.7%) of the respondents were girls while the remaining two hundred and eighty nine (47.3%) were boys. About half of the respondents (54.5%) were Muslims. Five hundred and twenty eight (86.4%) of the respondents were Yoruba. With respect to the class of the respondents, 40.1%, 34.5%, and 25.4% were in Primary 4, 5 and 6, respectively.

**Table 1.** Socio-demographic characteristics of primary school children who enrolled in the survey in Akinyele Local Government Area of Oyo State, Nigeria.

Characteristic	n	%
*Age (yrs)*		
7-9	611	26.4
10-13	450	73.6
*Sex*		
Male	289	47.3
Female	322	52.7
*Class*		
Primary 4	245	40.1
Primary 5	211	34.5
Primary 6	155	25.4
*Religion*		
Christian	278	45.5
Muslim	333	54.5
*Tribe*		
Yoruba	528	86.4
Hausa	33	5.4
Igbo	29	4.7
Others	21	3.4

### 3.1 Reported household ownership and source of insecticide-treated nets (ITNs)

Table 2 shows reported household ownership of insecticide-treated nets (ITN). The majority of the respondents (81.7%) reported having at least one ITN in their household. The number of ITNs owned varied. One hundred and thirty seven (27.5%) reported owning one ITN, (49.3%) reported had two ITNs while (23.2%) said they had three or more ITNs in their households.

**Table 2.** Reported household ownership of insecticide-treated nets (ITNs).

Variable	n	%
*ITNs in household*		
Yes	499	81.7
No	112	18.3
*# of ITNs in household*		
One	137	27.5
Two	246	49.3
Three or more	116	23.2
*Source of insecticide-treated nets*		
Donated by government	381	76.4
Purchased	68	13.6
Gift	46	9.2
Both government and purchase	3	0.6
Don’t know	1	0.2

The respondents identified the sources of the ITNs they had acquired in their households. Three hundred and eighty one (76.4%) said it was given to their parents by the local government, (13.6%) said their parents purchased the ITNs they had in their household, (9.2%) said it was a gift and (0.6%) said they got from the government and also purchased some while only one of the respondents did not know the source of the ITNs in their household.

### 3.2 Reported household use of insecticide-treated nets (ITNs)

Table 3 shows household use of ITNs as reported by the respondents. Four hundred and forty three (88.8%) of the respondents who had at least one ITN in their households stated that members of their household were currently using ITNs of which (67.7%) said they and members of their households slept under ITNs the night before the survey and (65.7%) of the respondents said that they and members of their households slept under ITNs daily. The results also showed that 51.6% of the respondents reported that their under-five brothers/sisters used ITNs. A few of the respondents (13.8 %) reported one person sleeping under one ITN, 42.9% reported two and 42.4% three or more persons sleeping under one ITN.

**Table 3.** Reported household use of insecticide-treated nets (ITNs).

Variable	n	%
*Ever used ITNs*		
Yes	447	89.6
No	52	10.4
*Currently using ITNs*		
Yes	443	88.8
No	56	11.2
*Frequency of use*		
Every day	291	65.7
At least once a week	50	11.3
Once in a while	102	23.0
*All household members use ITNs*		
Yes	102	23.0
No	341	77.0
*# of ITNs currently in use in household*		
One	192	43.3
Two	203	45.8
Three or more	48	10.8
*# of persons sharing ITNs*		
One	61	13.8
Two	190	42.9
Three or more	188	42.4
*ITN last used in household*		
Last night	300	67.7
A week ago	78	17.6
A month ago	37	8.4
A year ago	28	6.3
*ITN is hung on the wall*		
Yes	393	88.7
No	50	11.3
ITN users in the household*		
Mother	306	69.5
Father	228	51.8
Brother/Sister	300	68.2
Brother/Sister Under-five	227	51.6

*Multiple answers possible

### 3.3 Relationships between socio-demographic characteristics and reported household ownership of at least one ITN

Relationships between socio-demographic characteristics with school children’s report of household ownership of at least on ITN are shown in Table 4. More girls (84.5%) than boys (78.5%) reported household ownership of at least one ITN (p= 0.059). A similar proportion of respondents within the ages 10-13 (82.2%) reported household ownership of at least one ITN compared with those within ages 7-9 (81.1%) (p = 0.555). A significantly higher proportion of respondents in primary five (86.3%) compared with those in primary six (84.5%) and primary four (75.9%) (p=0.010) reported ownership of at least one ITN. A higher though not significantly different proportion of respondents who were Christians (84.2%) reported household ownership of at least one ITN compared with those who were Muslims (79.6%) (p=0.144).

**Table 4.** Relationship between socio-demographic characteristics and reported household ownership of at least one ITN.

Variable	Own at least one ITN					
	Yes		Yes		χ^2^	p value
	n	%	n	%		
*Sex*						
Male	227	78.5	62	21.5	3.572	0.059
Female	272	84.5	50	15.5		
*Age range*						
7-9	129	80.1	32	19.9	0.349	0.555
10-13	370	82.2	80	17.8		
*Class*						
Pry 4	186	75.9	59	24.1	9.217	0.010*
Pry 5	182	86.3	29	13.7		
Pry 6	131	84.5	24	15.5		
*Religion*						
Christian	234	84.2	44	15.8	2.135	0.144
Muslim	265	79.6	68	20.4		

## 4 Discussion

In this study, the ages of the respondents ranged from 7-13 years with a mean age of 10.5 ± 1.7 years. However, the age range used by different school-based studies varies. This age range was used in this study with the assumption that pupils within this age range are more likely than those of higher age range to have under-five children in their households. Of all the respondents, 65.3% indicated having children under-five in their households. The majority of the respondents were within the ages 10-13 years. This is comparable with a study among primary school children in which most of the respondents (70.0%) were 10-14 years of age [14]. There were more girls than boys in this study. This corroborates studies in Cross Rivers State, Nigeria that reported more girls (52.5%) than boys and in Ethiopia that recorded 65.7% of girls [15]. The majority of the respondents in this study were from the Yoruba ethnic group, this is because Akinyele Local Government Area (LGA) Oyo State, Nigeria is located in Southwestern Nigeria which is predominantly a Yoruba-speaking area.

The results presented show that more than a half of the respondents reported household ownership of at least one ITN. The findings from this study corroborates that of household surveys in Ekiti State, Southwest Nigeria which reported (95.3%) of households owning at least one ITN [16] and also in Kebbi State Nigeria which reported 75.3% of the respondents had at least one ITN [17]. However, this is far higher than the 44.2% bednet ownership reported by school children in Kenya [8] and the household surveys conducted in Nigeria, which reported 40.5% [13], 42.3% [1] for Southwest and 39.1% [1] for Oyo state Nigeria. These discrepancies may be because of the varying location of the Kenya study and because NDHS and MIS are national population surveys.

The respondents were able to identify the source of the available ITNs in their households. More than half of the respondents stated that it was obtained through mass distribution campaigns. This is in line with the Nigerian MIS [13], which stated that mass distribution campaigns are the main distribution channel for mosquito nets generally with (60.5%) of the mosquito nets in Southwest Nigeria obtained through mass distribution campaigns.

Studies have shown that ownership of ITNs does not guarantee the use of it. For instance, a study conducted in a Southwestern State in Nigeria reported 95.3% ownership and a utilisation rate of 59% [16]. Hence, it is important to monitor use among vulnerable groups and the population at large. The indicators for use of ITNs include the proportion of respondents who slept under an ITN the previous night, the proportion of under-five children who use ITNs, and the proportion of pregnant women who used an ITN during pregnancy. The findings from this study highlight that more than half of all the respondents who reported owning a net also reported that members of their households slept under an ITN the night before the survey. This is higher than the findings of household surveys conducted in Nigeria which stated household ITN/LLIN utilisation rate of 28.6% [1] and 16.0% [13]. Another key finding from this study is that half the respondents (50.4%) who indicated having ITNs in their households stated use of ITNs by under-five children. This is higher than the finding from household survey conducted in Nigeria, which reported 37.8% [1] and 16.2% [13] utilisation rate of IT-Ns/LLINs among under-five children.

Some studies have shown that reported household ownership of ITNs among school children could complement report from household heads. There was a statistically significant difference between class of respondents and reported ownership of ITNs. More of the respondents in primary five (86.3%) as compared to those in primary four and six reported household ownership of at least one ITN. This may be because of the age of the pupils in that class, most of the respondents within age 10-13 years were in primary five.

## 5 Conclusions

The pupils were able to report household ownership of insecticide treated nets (ITNs). They were able to state the numbers as well as the source of the ITNs in their households. The reported household use of ITNs was high and more than half of the respondents reported ITN use by under-five children in the household. Therefore, schoolchildren could be considered as a potential source of information for routine M&E of ownership and use of insecticide-treated bednets.
